# Pauling’s Conceptions of Hybridization and Resonance in Modern Quantum Chemistry

**DOI:** 10.3390/molecules26144110

**Published:** 2021-07-06

**Authors:** Eric D. Glendening, Frank Weinhold

**Affiliations:** 1 Department of Chemistry and Physics, Indiana State University, Terre Haute, IN 47809, USA; glendening@indstate.edu; 2 Theoretical Chemistry Institute and Department of Chemistry, University of Wisconsin-Madison, Madison, WI 53706, USA

**Keywords:** chemical bonding, directed hybridization, resonance delocalization, mesomerism, natural bond orbitals, natural resonance theory

## Abstract

We employ the tools of natural bond orbital (NBO) and natural resonance theory (NRT) analysis to demonstrate the robustness, consistency, and accuracy with which Linus Pauling’s qualitative conceptions of directional hybridization and resonance delocalization are manifested in all known variants of modern computational quantum chemistry methodology.

## 1. Introduction

The present authors proudly claim direct line of descent in the academic family tree of Linus Pauling. Senior author FW was an academic grandson (through *Doctorvater* E. B. Wilson, Jr. at Harvard University, 1963–1967), a faculty colleague (at Stanford University, 1974–1976), and a student of Pauling (in the 1975 Special Topics course on the valence bond theory of nuclear structure). Junior author EDG’s Ph.D studies with FW on chemical bonding [1,2,3] and resonance theory [4,5,6] at UW-Madison (1985–1991) were largely based on classic works of Pauling and Wilson [7,8] and conducted under their watchful eyes in photographic portraits that overlooked both the Theoretical Chemistry Institute (TCI) Lecture Room and FW’s office.

In the quarter-century following the first applications of quantum theory to chemical bonding [9,10], the powerful influence of Pauling’s valence bond (VB) formulation of hybridization [11,12] and resonance [13,14] theory could hardly be overstated. However, this influence waned as the rival molecular orbital (MO) formulation [15,16,17] achieved efficient numerical implementation [18,19,20,21,22,23] in the 1960s. Traditional VB theory was further weakened when Norbeck and Gallup [24] demonstrated that a strictly ab initio evaluation of the VB wavefunction for benzene gave results that were variationally inferior to MO theory and contradicted many semi-empirical VB assumptions of the time. Some limitations of the original VB formulation were removed in the self-consistent generalized GVB formulation of Goddard and co-workers [25,26] (and the related spin-coupled SCGVB variant [27]). However, the self-consistent orbital mixings tend to obscure interpretation of final GVB numerical results in terms of the VB-type initial guess. As density functional theoretic (DFT) and other MO-based methodologies advanced [28], VB-based methods were reduced to a niche role in quantum chemistry.

It is important to recognize that the validity of Pauling-type hybridization and resonance concepts is essentially independent of whether VB/GVB-type wavefunctions are computationally competitive. Pauling’s inspiration to “hybridize” free-atom spherical-harmonics to achieve improved bonding orbitals and compact wavefunctions was intended to rationalize the empirically known directionality of atomic valency (e.g., the *tetrahedral* carbon atom of van’t Hoff and LeBel [29,30,31]), as demanded by early structural studies [32,33,34]. As shown by Coulson [35], such directional hybrids (linear combinations of directionless free-atom *s*,*p*,*d*,… orbitals) can serve equally well as conceptual building blocks in MO and VB theory. Similarly, Pauling’s motivation to combine two (or more) Lewis-structural bonding patterns into a “resonance hybrid” was intended to rationalize the empirically known ambivalence of certain molecules (such as benzenoid species or practically any molecule containing allyl or amide groups) whose properties appear “intermediate” or “averaged” between the possible Lewis-structural bonding patterns that might be envisioned [36,37,38,39,40,41,42,43]. Pauling’s basic resonance concept was first expressed mathematically in terms of then-standard Heitler-London pair functions [9], which, in light of subsequent Norbeck-Gallup [24] and Coulson-Fischer [44] studies, can be recognized as a rather arbitrary and sub-optimal choice.

In more recent times, basic precepts of hybridization and resonance theory have been questioned or criticized on various grounds. Specific technical criticisms of hybridization theory are often based on uncritical application of Koopmans’-type approximations to interpret photoionization spectroscopy or pedagogical preference for VSEPR-type rationalizations of molecular structure [45] (but see contrarian views [46,47,48,49,50,51]). Early criticisms of resonance concepts were philosophically based on supposed conflicts with “realism” as perceived in dialectical materialism theory [52,53]. More specific technical criticisms of resonance (e.g., as a conceptual “unicorn” [54]) are often based on preferred use of energy decomposition analysis (EDA) methods that require a specific choice of “reference state” [55,56] for each interacting fragment (perforce eliminating resonance-type state-mixing in either fragment).

However, more general questioning of hybridization and resonance concepts can be attributed to the complex mathematical forms of modern wavefunctions and density functionals that no longer allow chemists to easily “see” the hybridization and resonance features that appear explicitly in VB-based formulations. Ironically, even some advocates of modern SCGVB theory have expressed skepticism about Pauling’s hybridization concepts [57,58], because the final orbital shapes no longer resemble localized VB-inspired forms. Related attempts to obtain directed hybrids of localized chemical bonding in the MO/DFT framework by transforming canonical MOs to localized LMO form [59,60,61,62,63] are similarly frustrated, because the localization procedure can be chosen rather arbitrarily to yield a virtually unlimited variety of orbital energies and shapes, with *no* effect on the calculated total energy or other measurable properties of the system.

All such conceptual dilemmas can be averted by adopting a uniform analysis of diverse wavefunctions in a common language of localized bonding constructs. For this purpose, we employ natural bond orbital (NBO) [64,65,66] and natural resonance theory (NRT) algorithms [67,68] that are implemented in a widely used program (currently *NBO 7.0* [69,70]). Although NBO/NRT methods rest on mathematical foundations somewhat beyond those usually discussed in introductory quantum chemistry, the *NBO 7.0* program is often integrated into the selfsame quantum chemistry program that generates the wavefunctions to be analyzed [71]. In other cases, the host quantum chemistry program that performs the wavefunction calculation is able to write the “wavefunction archive” (job.47) file that serves as input to a current NBO analysis program, either in stand-alone form or as included in other quantum chemistry program systems. The resulting NBO analysis allows consistent apples-to-apples comparisons of key bonding descriptors (such as atomic *s*,*p*,*d*,… composition of NBO-based bonding hybrids, or the NRT bond orders between atoms) no matter how diverse the wavefunctions to be compared.

In the present work, we employ a consistent protocol to obtain NBO/NRT descriptors of hybridization and resonance for prototype chemical species described at a wide variety of modern quantum chemistry levels, including GVB, DFT, and higher correlated methods. The results serve not only to show how comparison hybridization and resonance descriptors can be obtained from diverse wavefunctional forms, but also to exhibit their remarkable overall consistency with Pauling’s original intuitions dating back nearly nine decades. Pauling’s hybridization and resonance conceptions thereby seem to gain increasing theoretical support as the accuracy and applicability of modern quantum chemistry methods continue to improve.

## 2. Computational Methods

The present overview involves comparisons of many computational levels that are commonly identified in the arcane “method/basis” acronyms of modern computational quantum chemistry (see [28] for additional explanations and original references). In addition to RHF (restricted Hartree-Fock), the employed methods include B3LYP (Becke 3-parameter, Lee-Yang-Parr correlation functional variant of DFT theory), SCGVB, CAS (complete active space self-consistent-field), MP2 (2nd-order Møller-Plesset), and CCSD (coupled-cluster with single and double excitations). The basis set was chosen uniformly as “aVTZ” (Dunning-type augmented correlation-consistent valence triple zeta), but many other basis sets of higher or lower quality could be expected to give qualitatively similar numerical results. Geometries were optimized at the B3LYP/aVTZ or MP2/aVTZ level, as detailed below. Transition state searches and intrinsic reaction coordinate (IRC) calculations were performed at the B3LYP/aVTZ level. All calculations were completed with Gaussian-16 [72] except for single-point energy evaluations at the SCGVB and CAS levels [73,74], which were completed using Molpro [75,76,77]. Further numerical details of optimizations, IRC evaluation, and NRT keyword settings are described in Supplementary Materials.

## 3. Directional Hybridization

Hybridization of atomic orbitals is a central concept in modern chemical bonding theory. As described by Pauling [11] and Slater [12], the mixing of valence *s* and *p* orbitals at a tetrahedral carbon atom facilitates electron-pair bonding by forming four equivalent hybrids that are directed toward the vertices of the regular tetrahedron. More generally, valence orbitals of any main group atom can undergo hybridization in a molecular environment to give a set of four directed hybrids (*i* = 1–4)

(1) $$h_{i}=\frac{1}{\sqrt{1+\lambda_{i}}}\left(s+\sqrt{\lambda_{i}}p_{\theta_{i}}\right)$$

of $sp^{\lambda_{i}}$ character, where $p_{\theta_{i}}$ is a valence *p* orbital aligned with direction $\theta_{i}$ and the hybridization parameter $\lambda_{i}$ can range from 0 (pure *s*) to ∞ (pure *p*). We assume here that mixing is limited to orbitals of *s* and *p* symmetry only, which is typical for normal-valent main group atoms (where *d*-character in these hybrids is generally less than 0.2%). Conservation of valence *s*- and *p*-character requires that

(2) $$\sum_{i}\frac{1}{1+\lambda_{i}}=1$$

(3) $$\sum_{i}\frac{\lambda_{i}}{1+\lambda_{i}}=3$$

where $1/\left(1+\lambda_{i}\right)$ and $\lambda_{i}/\left(1+\lambda_{i}\right)$, respectively, represent the fractional *s*- and *p*-character of the *i*th hybrid and the summations run over all four hybrids. These conservation expressions are only satisfied for a mutually orthogonal set of atomic hybrids.

Before illustrating hybridization in NBO analysis, let us briefly review the procedure that yields the “natural hybrid orbitals” (NHOs). NBO analysis begins with the first-order reduced density matrix **Γ** for any *N*-electron wavefunction *ψ*(1,2, …,*N*). This matrix has elements

(4) $$\Gamma_{ij}=\int \chi_{i}^{*}\left(1\right)\;\hat{\Gamma }\left(1|1^{\prime }\right)\chi_{j}\left(1^{\prime }\right)\;d1\;d1^{\prime }$$

for atom-centered basis functions {$\chi_{k}$} and density (integral-) operator $\;\hat{\Gamma }\left(1|1^{\prime }\right)$,

(5) $$\;\hat{\Gamma }\left(1|1^{\prime }\right)=N\int \psi \left(1,\;2,\;\ldots ,\;N\right)\;\psi^{*}\left(1^{\prime },\;2,\;\ldots ,\;N\right)\;d2\ldots dN$$

We assume here that the density matrix is represented in an orthogonal basis. If the basis functions are instead non-orthogonal, as is usually the case, the density is first transformed to an orthogonal “natural atomic orbital” (NAO) representation, the details of which are described elsewhere [78]. NBO analysis then seeks the set of localized one- and two-center orbitals, the natural bond orbitals (NBOs), that best represent the electron density. The NHOs are the atomic components of these NBOs.

NBOs are obtained from eigenvectors of one- and two-center blocks of the density matrix. The NBO search procedure initially searches one-center blocks, selecting all eigenvectors having occupancies (eigenvalues) that exceed threshold (initially 1.90*e*, the “occupancy threshold”). These vectors are identified as atomic core and lone pair orbitals, and the density associated with these functions is projected from the density matrix. The procedure next searches two-center blocks of the projected density matrix, selecting eigenvectors that again have occupancies exceeding threshold. These two-center vectors are generally non-orthogonal, and those vectors that overlap considerably (squared-overlap exceeding 0.70) at common centers are eliminated. The remaining vectors are orthogonalized using an occupancy-weighted symmetry orthogonalization procedure [78]. This yields the set of orthogonal, two-center orbitals (the bonds), each A–B bonding orbital

(6) $$\Omega_{\mathrm{AB}}=c_{A}h_{A}+c_{B}h_{B}$$

represented as a linear combination of atomic bonding hybrids, *h*_A_, *h*_B_, with polarization coefficients *c*_A_, *c*_B_. The set of NHOs includes all one-center NBOs and all hybrids, *h*_A_, *h*_B_, of the two-center NBOs, along with extra-valence Rydberg functions that complete the span of the basis set. The one- and two-center NBOs together often account for over 99.9% of the calculated electron density.

**Figure 1:**
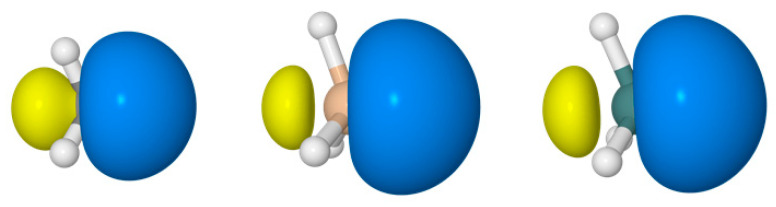
Pre-orthogonal bonding hybrids for CH_4_ (**left**), SiH_4_ (**middle**), and GeH_4_ (**right**).

Figure 1 shows representative bonding hybrids for the central atoms of CH_4_, SiH_4_, and GeH_4_. The orbitals depicted in this figure are “pre-orthogonal” because although they are orthogonal to all other hybrids on the central atom, each can strongly overlap the 1 *s* orbital of the adjacent H atom to which the hybrid is directed.

NHO character is found to be largely independent of the ab initio or density functional method employed, as illustrated for the 15 main group hydrides of Table 1. The *p*-character of the bonding hybrids is reported for a range of computational methods, for densities calculated at the single-determinantal uncorrelated (RHF), multi-determinantal correlated (SCGVB, CAS), and single-reference correlated (MP2, CCSD) levels, and with density functional theory (B3LYP), all at fixed MP2/aVTZ optimized geometries. For each hydride, the *p*-character varies weakly across the series of densities. Even for HBr, which exhibits the largest *λ* variation (from 6.82 at the RHF level to 7.81 for SCGVB), the percent *p*-character changes by only 1.4% (from 87.2% to 88.6%). Note specifically that the SCGVB hybrid descriptors of Table 1 are generally in line with the near-Pauling results that are found both at higher and lower computational levels, contrary to the conclusions of [57,58]. Thus, the NBO user can be confident that the hybrid description offered at one level of theory will be largely consistent with that obtained using nearly any other level, particularly for densities from correlated or density functional calculations.

**Figure 2:**
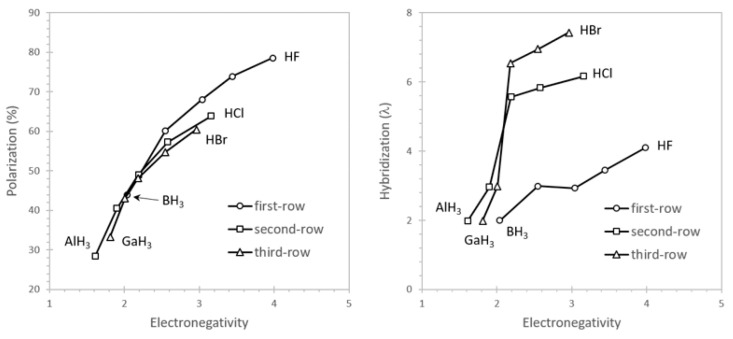
Polarization (*c*_X_^2^) and hybridization (*λ*) of the X–H bonds of normal-valent hydrides as a function of the Pauling electronegativity of the main group atom (MP2/aVTZ).

Figure 2 shows the character of the X–H bonds, including bond polarization (*c*_X_^2^) and hybridization (*λ*) of the main group atom. As the electronegativity of X increases, the bond increasingly polarizes and the bonding hybrid gains *p*-character, as anticipated by Bent’s rule [79,80]. The Group 13 hydrides (XH_3_, X = B, Al, Ga) have trigonal planar geometries so that the central atoms are essentially *sp*^2^ hybridized (67% *p*), as confirmed by the NHOs. Similarly, the Group 14 hydrides (XH_4_, X = C, Si, Ge) are tetrahedral with *sp*^3^-like hybrids (75% *p*), consistent with Pauling’s original inferences from molecular symmetry.

The Group 17 hydrides reveal the highest *p*-character, with *λ* = 4.10 for HF, *λ* = 6.17 for HCl, and *λ* = 7.43 for HBr. Elevated *p*-character arises as *s*-character shifts from the bonding hybrid into a lone pair, thereby stabilizing the molecule. To illustrate, consider HF. The F atom has four valence orbitals, including the bonding hybrid and three lone pairs. Two of the lone pairs are π-type orbitals (unhybridized 2*p*) directed along vectors that are orthogonal to the line-of-centers. The third lone pair is σ-type, directed along the line-of-centers but away from the H atom. The latter orbital is *sp*^0.24^ hybridized (80.4% *s*), so by conservation of hybrid character, only 19.6% *s*-character is available for the bonding hybrid (*sp*^4.10^). The lone pair is essentially doubly occupied (1.982*e*), whereas the bonding hybrid has an occupancy (1.553*e*) considerably less than two electrons. HF is therefore stabilized because the higher occupancy lone pair has enhanced *s*-character, leaving limited *s*-character for the bonding hybrid. The bonding hybrids for the Group 15 and 16 hydrides have similarly elevated *p*-character—*s*-character concentrates in a lone pair of the central atom [79]. More general vertical (size-dependent) aspects of Bent’s rule are discussed elsewhere [81].

Group 15 and 16 hydrides may exhibit some bond bending if the central atom hybrids deviate from the X–H line-of-centers. In contrast, there is no bending in the Group 13 (XH_3_, *D*_3h_), 14 (XH_4_, *T*_d_), and 17 (HX, *C*_∞v_) hydrides because symmetry requires alignment with the line-of-centers. Table 2 compares the MP2 optimized bond angles of the Group 15 and 16 hydrides with two measures of inter-hybrid angle. The first of these, $\alpha =\cos^{-1}\left(1/\lambda \right)$, is the angle between a pair of *sp*^λ^-hybridized orbitals, equivalent to the angle between the $p_{\theta_{i}}$ orbitals [cf Equation (1)] for the hybrid pair. This measure assumes no contribution from polarization (*d*, *f*, etc.) functions. A second measure, β, is the angle subtended by the line segment that connects the points of maximum amplitude for the pair of bonding hybrids. We see in Table 2 that the inter-hybrid angles are consistently several degrees larger than the inter-nuclear bond angle. That the α angles are particularly large is not surprising because this measure ignores polarization effects. The β angles are somewhat smaller than α because *d*-character (typically approximately 0.2% of the hybrid) allows for the polarization of the hybrids, thereby shifting the amplitude maxima to somewhat more acute angles. We find that the β values are usually in fairly good accord with the optimized bond angles, except in cyclic species with appreciable ring-strain (e.g., cyclopropane).

All the foregoing results are qualitatively consistent with the intuitions that animated Pauling’s original conception of hybridization, long before the availability of respectably accurate wavefunctions by current standards. Accordingly, these hybrid intuitions continue to warrant central focus in chemical pedagogy, contrary to the conclusions expressed by Grushow [45].

## 4. Resonance Delocalization

As mentioned above, Pauling’s original formulation of the theory of resonance in chemistry [13] was grounded in mesomerism concepts [36,37,38,39,40,41,42,43] that could be rationalized and broadly extended in the abstract language and mathematical constructs of quantum mechanics. However, Pauling’s powerful resonance-based intuitions were largely honed by encyclopedic familiarity with available chemical structural data, rather than then-available VB formulations (later shown to be significantly flawed [24]). As recounted by Eisenberg [82], Pauling’s celebrated discovery of the α-helix was inspired by folding a cut-out paper model of a protein chain with resonance-enforced planarity at each amide group, a crude “analog device” to effectively bypass numerical VB-based modeling. In the present section, we employ NRT analysis to re-examine Pauling’s resonance-type concepts of amide structure and reactivity in the framework of modern quantum-chemical computations for a simple amide tautomerization reaction.

In the NRT formulation [4], resonance weightings {*w*_α_} are obtained from convex-type (*w*_α_ ≥ 0, ∑_α_ *w*_α_ = 1) expansion of the *electron density* operator, with weightings chosen to optimally approximate the full quantum-chemical density operator ${\;\hat{\Gamma }}_{\mathrm{QC}}$ for the chosen ab initio or density functional calculation. The corresponding resonance-type ${\;\hat{\Gamma }}_{\mathrm{NRT}}$ expansion

(7) $${\;\hat{\Gamma }}_{\mathrm{NRT}}=\sum_{\alpha }w_{\alpha }{\;\hat{\Gamma }}_{\alpha }$$

is expressed as a weighted sum of localized density operators ${\;\hat{\Gamma }}_{\alpha }$, one operator for each idealized localized bonding pattern α contributing to the resonance hybrid. Resonance weights, *w*_α_, are variationally optimized subject to normalization and positivity constraints

(8) $$\sum_{\alpha }w_{\alpha }=1;\;w_{\alpha }\geq 0$$

by minimizing the Frobenius norm

(9) $$\underset{w_{\alpha }}{\min}\|\Gamma_{\mathrm{QC}}-\Gamma_{\mathrm{NRT}}\|.$$

An efficient and parallelized implementation of NRT is available in *NBO 7.0* [69]. In addition to reporting the details of the resonance hybrid (weights and structures), NRT calculates “natural bond orders”

(10) $$b_{\mathrm{AB}}=\sum_{\alpha }w_{\alpha }b_{\mathrm{AB}}^{\left(\alpha \right)}$$

where $b_{\mathrm{AB}}^{\left(\alpha \right)}$ is the integer bond order of the A–B atom pair of resonance structure α.

**Figure 3:**
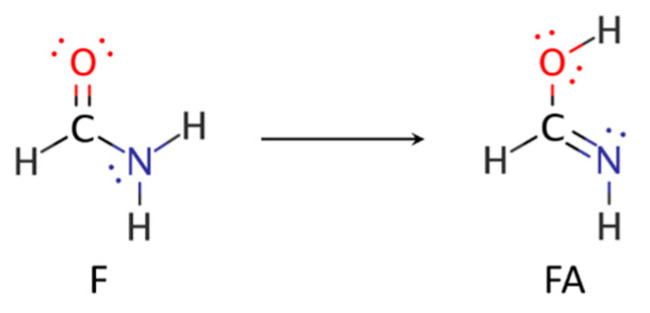
Schematic bonding patterns for tautomeric isomerization of formamide (F) to formimidic acid (FA).

We illustrate application of NRT by considering formamide (F)-formimidic acid (FA) tautomerization (Figure 3). Formamide is the simplest naturally occurring molecule that features the N-C=O peptide bond. Its conversion to formimidic acid is catalyzed by solvent molecules or by another formamide molecule, but we only examine here the uncatalyzed intramolecular reaction that proceeds via a simple 1,3-proton transfer mechanism.

**Figure 4:**
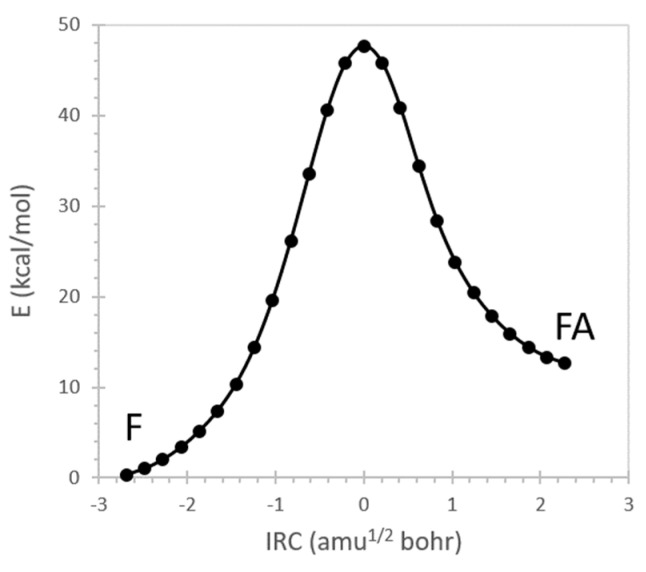
B3LYP/aVTZ energy profile for the tautomerization of formamide (F) to formimidic acid (FA). IRC = 0 corresponds to the transition state.

Figure 4 shows the B3LYP/aVTZ energy profile for tautomerization. Formimidic acid is 12.3 kcal/mol less stable than formamide and is separated from formamide by a 47.7 kcal/mol barrier. The barrier is consistent with the 47.4 kcal/mol estimate calculated by Hazra and Chakraborty [83] at the MP2/6-311++G ** level.

NRT analysis of formamide yields the resonance hybrid of Table 3. Formamide is well represented by just four resonance structures that collectively describe 99.3% of the resonance expansion. The molecule is sufficiently delocalized that the dominant resonance form (**F1**, the “natural Lewis structure”) only contributes 43.8% of the resonance hybrid. The leading secondary structure, the charge-transfer form **F2** at 33.7%, stems from strong π-type resonance as electron density from the N lone pair, *n*_N_, delocalizes into the π_CO_ * antibond. An image of this donor–acceptor interaction in Table 3 shows significant orbital overlap between the C and N atoms (on the right) that lends considerable double-bond character to the CN bond, while electron transfer into the π_CO_ * antibond (on the left) acts to reduce CO double-bond character. These effects on bond order are consistent with the mixing of the **F2** structure into the resonance hybrid. Perturbative analysis of the Kohn-Sham matrix suggests that the *n*_N_ → π_CO_ * interaction alone stabilizes formamide by about 62 kcal/mol. Two additional interactions, both involving σ-type delocalization of electrons from an O lone pair are somewhat weaker (at 26.2 and 22.8 kcal/mol) and result in smaller, although still significant, contributions to the resonance expansion from structures **F3** and **F4**.

Table 4 shows the corresponding analysis for formimidic acid. Like formamide, formimidic acid is fairly well described by four resonance structures, with weights totaling 98.2%. The Lewis structure (**FA1**) dominates the resonance expansion at 64.2%, and the leading charge-transfer form (**FA2**) at 20.2% arises from π-type delocalization of an O lone pair, *n*_O_, into the π_CN_ * antibond. This charge-transfer interaction is stabilizing by about 40.4 kcal/mol. Two weaker σ-type delocalizations, involving the N lone pair and NH bond, contribute about 14% of the resonance hybrid. The resonance expansion clearly suggests that resonance delocalization effects are somewhat weaker in formimidic acid than in formamide, which probably accounts for the greater stability (by ~12 kcal/mol) of the latter tautomer.

**Figure 5:**
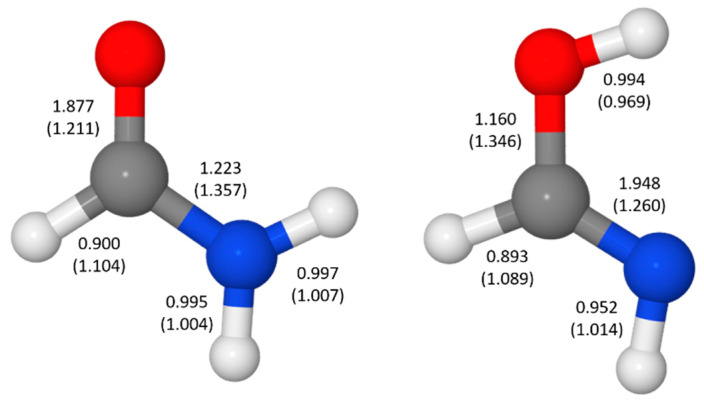
Natural bond orders and optimized bond lengths (in parentheses, in Å) for formamide and formimidic acid.

Natural bond orders for formamide and formimidic acid are shown in Figure 5, along with the optimized bond lengths. These bond orders are weighted-averages of the integer bond orders for the structures **F1**–**F4** of Table 3 and **FA1**–**FA4** of Table 4, respectively. Ignoring the proton transfer, the principal geometry changes during tautomerization are the lengthening of the CO bond (by 0.135 Å) and shortening of the CN bond (by 0.097 Å). These changes result from the loss of CO double-bond character (bond order decreasing from 1.877 to 1.160) and gain of CN double-bond character (increasing from 1.223 to 1.948) as the resonance description morphs from mostly **F1** to predominantly **FA1**.

**Figure 6:**
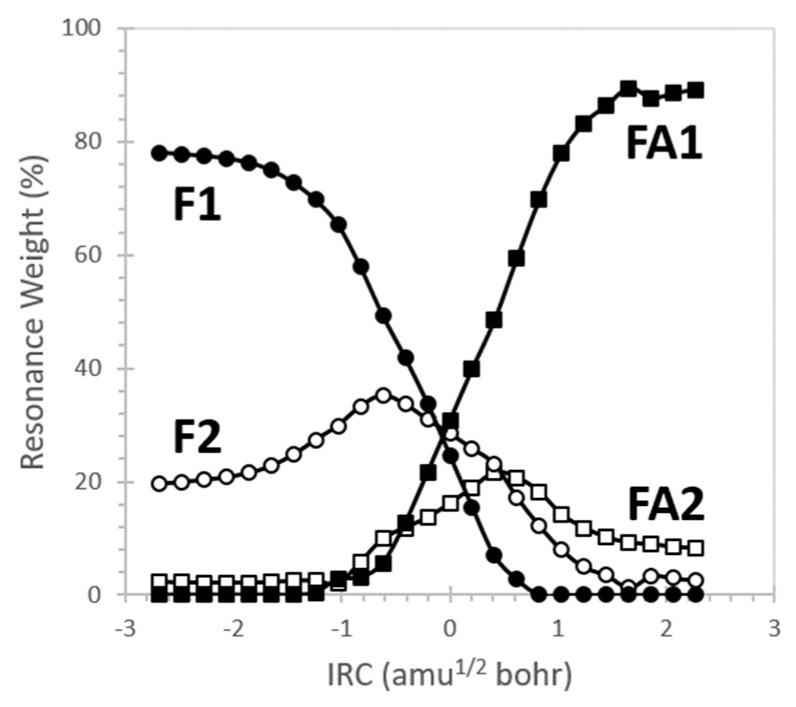
Variation in resonance weights along the tautomerization IRC.

We examined tautomerization more fully by performing NRT analysis at geometries across the reaction profile of Figure 4. To simplify the analysis, we limited the NRT expansion to only four resonance forms, including the two dominant structures of formamide (**F1** and **F2**) and the two dominant structures of formimidic acid (**FA1** and **FA2**) [84]. These four structures alone constitute the minimal set required to simultaneously describe bond breaking/formation during proton transfer and resonance effects in the π system (neglecting weaker σ-type resonance contributions). Figure 6 shows the dependence of the resonance weights on reaction coordinate.

F → FA conversion begins with formamide electron density described by **F1** and **F2** in roughly 80%:20% proportion. As proton transfer begins π resonance strengthens as the **F2** contribution increases. Note that **F2** has the same N=C-O bonding pattern as the product Lewis structure **FA1**, although the latter only begins to contribute importantly to the resonance hybrid within close proximity to the transition state (IRC = 0). The transition state is strongly delocalized with nearly equal contributions (~28%) from **F1**, **F2**, and **FA1**. When the reaction is complete, the formimidic acid is roughly 90% **FA1** and 10% **FA2**.

**Figure 7:**
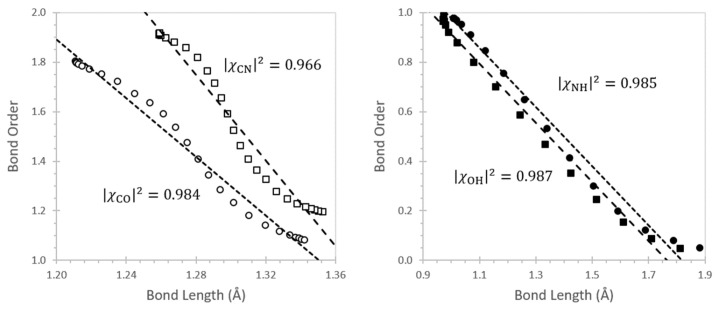
Bond order–bond length correlations for CO (circles), CN (squares), OH (filled circles), and NH (filled squares) bonds, showing the least-squares regression line and corresponding Pearson |χ|^2^ correlation coefficient for each bond type.

Figure 7 shows the correlation of natural bond order with bond length for geometries across the IRC. The correlations reveal slight S-shaped curvatures, or more specifically, near-perfect linear correlations around each integer (single dominant NLS) or half-integer (two-state bond-shift [85]) bond order with connecting curvatures to accommodate the slightly different slopes of different bond types, but their essential linearity is suggested by the robust |χ|^2^ coefficients. Such correlations strongly support the useful predictive associations of NRT bond orders with experimentally measurable quantities, consistent with well-known empirical relationships connecting a variety of bond properties, including bond lengths [86,87,88], bond energies [89,90,91,92], IR vibration frequencies [93,94], and NMR spin-spin coupling constants [95].

**Figure 8:**
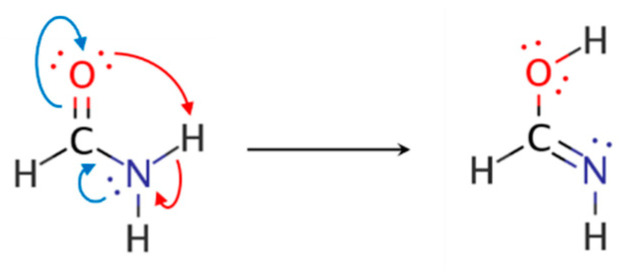
Curly-arrow depiction of resonance-type electronic delocalizations in formamide tautomerization.

Variations in the NRT weights for the four resonance structures across the IRC, as well as concomitant changes in natural bond orders, are entirely consistent with the electron-pushing, curly-arrow representation that the bench chemist would use to depict the reaction mechanism (Figure 8). Red arrows correspond to the bond/lone pair rearrangement associated with proton migration, and blue arrows represent the change in π electron distribution of the peptide bond.

With this simple example, we have shown that NRT analysis provides a tool for easily obtaining compact and chemical intuitive descriptors of molecular structure and reactivity that are fully consistent with the prescient mesomerism/resonance insights of Pauling, Robinson, Ingold, and other bonding pioneers, dating back to the pre-quantum mechanical era. Similar to the hybridization results presented above, the present B3LYP/aVTZ results are fully representative of those obtained from numerically complex quantum chemical wavefunctions at any reasonably current computational level.

## 5. Summary and Conclusions

Contrary to skepticism that is sometimes expressed [45,54,58], we believe that the present results confirm the essential correctness and usefulness of Pauling’s hybridization and resonance concepts, as consistently found in NBO/NRT analysis of wavefunctions from the best currently available quantum chemical methods. If anything, improved quantitative accuracy of the wavefunction tends to enhance admiration of Pauling’s powerful intuitions, developed long before numerically reliable solutions of Schrödinger’s equation became routinely available.

In closing this tribute, it may be appropriate to relate that E. Bright Wilson considered John von Neumann and Linus Pauling to be the only two authentic geniuses he ever met. Elite company indeed!

## Figures and Tables

**Table 1:** Hybrid *p*-character (*λ*) of X–H bonding hybrids for the first-, second-, and third-row hydrides ^a^.

	RHF	B3LYP	SCGVB	CAS	MP2	CCSD
BH_3_	2.00	2.00	2.00	2.00	2.00	2.00
CH_4_	2.99	3.00	2.99	2.99	2.99	2.99
NH_3_	2.89	2.90	2.96	2.99	2.93	2.94
H_2_O	3.24	3.31	3.42	3.47	3.45	3.44
HF	3.69	3.86	4.05	4.09	4.10	4.08
AlH_3_	1.97	1.98	1.98	1.98	1.98	1.98
SiH_4_	2.96	2.98	2.96	2.96	2.96	2.96
PH_3_	5.38	5.72	5.49	5.60	5.57	5.67
H_2_S	5.55	5.83	5.88	6.00	5.83	5.93
HCl	5.79	5.93	6.46	6.55	6.17	6.24
GaH_3_	1.99	2.00	1.99	1.99	1.99	1.99
GeH_4_	2.99	3.00	2.99	2.99	2.99	2.99
AsH_3_	6.14	6.91	6.37	6.47	6.54	6.64
H_2_Se	6.43	7.11	7.00	7.05	6.95	7.06
HBr	6.82	7.27	7.81	7.80	7.43	7.52

^a^ aVTZ values calculated at MP2/aVTZ optimized geometries.

**Table 2:** Bond angles (∠HXH) and inter-hybrid angles (α and β) of the Group 15 and 16 hydrides ^a^.

	∠HXH	α	β
NH_3_	106.8	110.0	107.9
H_2_O	104.1	106.8	104.7
PH_3_	93.6	100.3	98.0
H_2_S	92.2	99.9	95.6
AsH_3_	92.5	98.8	98.8
H_2_Se	91.1	98.3	96.8

^a^ MP2/aVTZ values in degrees.

**Table 3:** NRT resonance hybrid of formamide ^a^.

#	Structure	Weight (%)	Donor–Acceptor Interaction ^b^	
**F1**	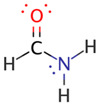	43.8		
**F2**	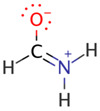	33.7	*n*_N_→*π*_CO_ * (61.9)	* 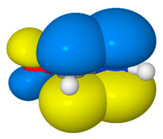 *
**F3**	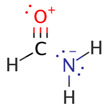	12.1	*n*_O_→*σ*_CN_ * (26.2)	* 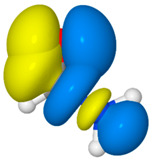 *
**F4**	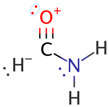	9.7	*n*_O_→*σ*_CH_ * (22.8)	* 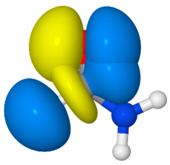 *

^a^ B3LYP/aVTZ values. ^b^ Donor–acceptor interactions of the natural Lewis structure (**F1**) that are isomorphic with the secondary resonance structures. Values in parentheses are second-order estimates of the interaction strength in kcal/mol. Images depict the favorable overlaps of the donor–acceptor NBO pairs.

**Table 4:** NRT resonance hybrid of formimidic acid ^a^.

#	Structure	Weight (%)	Donor–Acceptor Interaction ^b^	
**FA1**	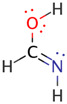	64.2		
**FA2**	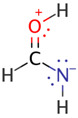	20.2	*n*_O_→*π*_CN_ * (40.4)	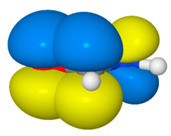
**FA3**	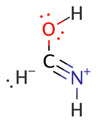	10.1	*n*_N_→*σ*_CH_ * (10.7)	* 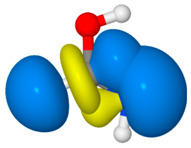 *
**FA4**	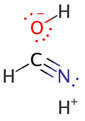	3.7	*σ*_NH_→*σ*_CO_ * (10.6)	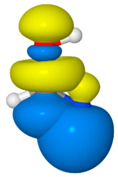

^a^ B3LYP/aVTZ values. ^b^ Donor–acceptor interactions of the natural Lewis structure (**FA1**) that are isomorphic with the secondary resonance structures. Values in parentheses are second-order estimates of the interaction strength in kcal/mol. Images depict the favorable overlaps of the donor–acceptor NBO pairs.

## Data Availability

The data presented in this study are available in Supplementary Materials.

## Supplementary Materials

Supplementary Materials are available online, including (i) Gaussian input files for all optimized geometries and the formamide–formimidic acid transition state, (ii) all IRC geometries, and (iii) a sample Gaussian input file, with NBO/NRT input for one of the IRC geometries.
