# Supplementary material for: Pauling’s Conceptions of Hybridization and Resonance in Modern Quantum Chemistry
Source: Molecules. 2021 Jul 6;26(14):4110. doi: 10.3390/molecules26144110 (PMC8303469; doi:10.3390/molecules26144110)
Supplement: Supplementary file 1 [file molecules-26-04110-s001.zip › molecules-1267467-SI.pdf]

Supplementary Material for:

Pauling's Conceptions of Hybridization and Resonance in Modern Quantum Chemistry

Eric D. Glendening[1] and Frank Weinhold[2,*]

[1] Department of Chemistry and Physics, Indiana State University, Terre Haute, IN 47809; glendening@indstate.edu
[2] Theoretical Chemistry Institute and Department of Chemistry, University of Wisconsin-Madison, Madison, WI 53706; weinhold@chem.wisc.edu
* Correspondence: weinhold@chem.wisc.edu

3. Directional Hybridization

The following is a single, multi-step Gaussian-16 input file that completes frequency calculations and NBO7 analysis for each of the MP2/aug-cc-pVTZ optimized geometries of the main-group hydrides of Table 1.

```
#mp2/aug-cc-pVTZ density=mp2 freq pop=nbo7read

BH3...mp2/avtz

0   1
B      0.000000    0.000000    0.000000
H      0.000000    1.187202    0.000000
H      1.028147   -0.593601    0.000000
H     -1.028147   -0.593601    0.000000

$nbo archive file=bh3-mp2 $end

--Link1--
#mp2/aug-cc-pVTZ density=mp2 freq pop=nbo7read

CH4...mp2/avtz

0   1
C      0.000000    0.000000    0.000000
H      0.627106    0.627106    0.627106
H     -0.627106   -0.627106    0.627106
H     -0.627106    0.627106   -0.627106
H      0.627106   -0.627106   -0.627106

$nbo archive file=ch4-mp2 $end

--Link1--
#mp2/aug-cc-pVTZ density=mp2 freq pop=nbo7read

NH3...mp2/avtz

0   1
N      0.000000    0.000000    0.114012
H      0.000000    0.938063   -0.266027
H      0.812387   -0.469032   -0.266027
```

```
H    -0.812387   -0.469032   -0.266027

$nbo archive file=nh3-mp2 $end

--Link1--
#mp2/aug-cc-pVTZ density=mp2 freq pop=nbo7read

H2O...mp2/avtz

0  1
O      0.000000    0.000000    0.118232
H      0.000000    0.758133   -0.472927
H      0.000000   -0.758133   -0.472927

$nbo archive file=h2o-mp2 $end

--Link1--
#mp2/aug-cc-pVTZ density=mp2 freq pop=nbo7read

HF...mp2/avtz

0  1
F      0.000000    0.000000    0.092184
H      0.000000    0.000000   -0.829654

$nbo archive file=hf-mp2 $end

--Link1--
#mp2/aug-cc-pVTZ density=mp2 freq pop=nbo7read

AlH3...mp2/avtz

0  1
Al     0.000000    0.000000    0.000000
H      0.000000    1.579872    0.000000
H     -1.368209   -0.789936    0.000000
H      1.368209   -0.789936    0.000000

$nbo archive file=alh3-mp2 $end

--Link1--
#mp2/aug-cc-pVTZ density=mp2 freq pop=nbo7read

SiH4...mp2/avtz

0  1
Si     0.000000    0.000000    0.000000
H      0.853257    0.853257    0.853257
H     -0.853257   -0.853257    0.853257
H     -0.853257    0.853257   -0.853257
H      0.853257   -0.853257   -0.853257

$nbo archive file=sih4-mp2 $end
```

```
--Link1--
#mp2/aug-cc-pVTZ density=mp2 freq pop=nbo7read

PH3...mp2/avtz

0  1
P      0.000000    0.000000    0.127012
H      0.000000    1.189254   -0.635061
H      1.029924   -0.594627   -0.635061
H     -1.029924   -0.594627   -0.635061

$nbo archive file=ph3-mp2 $end

--Link1--
#mp2/aug-cc-pVTZ density=mp2 freq pop=nbo7read

H2S...mp2/avtz

0  1
S      0.000000    0.000000    0.102929
H      0.000000    0.963093   -0.823429
H      0.000000   -0.963093   -0.823429

$nbo archive file=h2s-mp2 $end

--Link1--
#mp2/aug-cc-pVTZ density=mp2 freq pop=nbo7read

HCl...mp2/avtz

0  1
Cl     0.000000    0.000000    0.070819
H      0.000000    0.000000   -1.203922

$nbo archive file=hcl-mp2 $end

--Link1--
#mp2/aug-cc-pVTZ density=mp2 freq pop=nbo7read

GaH3...mp2/avtz

0  1
Ga     0.000000    0.000000    0.000000
H      0.000000    1.548180    0.000000
H     -1.340763   -0.774090    0.000000
H      1.340763   -0.774090    0.000000

$nbo archive file=gah3-mp2 $end

--Link1--
#mp2/aug-cc-pVTZ density=mp2 freq pop=nbo7read

GeH4...mp2/avtz
```

```
0  1
Ge    0.000000    0.000000    0.000000
H     0.872835    0.872835    0.872835
H    -0.872835   -0.872835    0.872835
H    -0.872835    0.872835   -0.872835
H     0.872835   -0.872835   -0.872835

$nbo archive file=geh4-mp2 $end

--Link1--
#mp2/aug-cc-pVTZ density=mp2 freq pop=nbo7read

AsH3...mp2/avtz

0  1
As    0.000000    0.000000    0.068882
H     0.000000    1.250750   -0.757701
H     1.083181   -0.625375   -0.757701
H    -1.083181   -0.625375   -0.757701

$nbo archive file=ash3-mp2 $end

--Link1--
#mp2/aug-cc-pVTZ density=mp2 freq pop=nbo7read

H2Se...mp2/avtz

0  1
Se    0.000000    0.000000    0.056417
H     0.000000    1.035567   -0.959086
H     0.000000   -1.035567   -0.959086

$nbo archive file=h2se-mp2 $end

--Link1--
#mp2/aug-cc-pVTZ density=mp2 freq pop=nbo7read

HBr...mp2/avtz

0  1
Br    0.000000    0.000000    0.039072
H     0.000000    0.000000   -1.367534

$nbo archive file=hbr-mp2 $end
```

4. Resonance Delocalization

The following is a single, multi-step Gaussian-16 input file that completes frequency calculations and NBO7/NRT analysis for B3LYP/aug-cc-pVTZ optimized geometries of formamide, formimidic acid, and the transition state.

```
#b3lyp/aug-cc-pVTZ freq pop=nbo7read

Formamide...b3lyp/avtz

0  1
C     0.000000    0.418861    0.000000
O     1.196495    0.233360    0.000000
N    -0.937329   -0.562322    0.000000
H    -0.446298    1.428425    0.000000
H    -0.646688   -1.526074    0.000000
H    -1.917677   -0.346147    0.000000

$nbo nrt archive file=formamide $end

--Link1--
#b3lyp/aug-cc-pVTZ freq pop=nbo7read

Formimidic acid...b3lyp/avtz

0  1
C     0.000000    0.416598    0.000000
O    -1.110473   -0.343364    0.000000
N     1.156615   -0.083827    0.000000
H    -0.263395    1.473261    0.000000
H    -0.825669   -1.270012    0.000000
H     1.876541    0.630866    0.000000

$nbo nrt archive file=formimidic-acid $end

--Link1--
#b3lyp/aug-cc-pVTZ freq pop=nbo7read

Formamide -> Formimidic acid TS...b3lyp/avtz

0  1
C     0.000000    0.509746    0.000000
O    -1.099333   -0.147985    0.000000
N     0.988617   -0.331290    0.000000
H     0.036873    1.595627    0.000000
H    -0.131137   -1.064739    0.000000
H     1.968609   -0.086449    0.000000

$nbo nrt archive file=ts $end
```

The following is a sample Gaussian-16 input file showing the set of $NBO keylist keywords used to complete NRT analysis of the IRC geometry corresponding to the leftmost point of Figure 3. The resonance hybrid is restricted to the four bonding patterns specified in the $NRTSTR keylist input. NRT analysis yields the weights of the leftmost points of Figure 5. See the *NBO 7.0 Program Manual* [37] for a description of NBO/NRT keywords.

```
#b3lyp/aug-cc-pVTZ pop=nbo7read

Point= -130  IRC= -2.68835  Energy=  -169.969711361 a.u. ###

0  1
C    -0.023518    0.433276    0.000000
O    -1.135351   -0.046133    0.000000
N     1.089975   -0.334942    0.000000
H     0.174323    1.518274    0.000000
H     0.960009   -1.333351    0.000000
H     2.020075    0.042092    0.000000

$nbo
  nrte2=off nrtnbi=off nrtdeg=off nrtlst=0.1 thrmin=1.1 prjthr=0.1
$end
$nrtstr
  str                    ! Structure F1
    lone 2 2 3 1 end
    bond d 1 2 s 1 3 s 1 4 s 3 5 s 3 6 end
  end
  str                    ! Structure F2
    lone 2 3 end
    bond s 1 2 d 1 3 s 1 4 s 3 5 s 3 6 end
  end
  str                    ! Structure FA1
    lone 2 2 3 1 end
    bond s 1 2 d 1 3 s 1 4 s 2 5 s 3 6 end
  end
  str                    ! Structure FA2
    lone 2 1 3 2 end
    bond d 1 2 s 1 3 s 1 4 s 2 5 s 3 6 end
  end
$end
```

The following is a list (in XYZ file format) of the IRC geometries of Figure 3.

```
  6
Point= -130  IRC= -2.68835  Energy=  -169.969711361 a.u.
C    -0.023518    0.433276    0.000000
O    -1.135351   -0.046133    0.000000
N     1.089975   -0.334942    0.000000
H     0.174323    1.518274    0.000000
H     0.960009   -1.333351    0.000000
H     2.020075    0.042092    0.000000
  6
Point= -120  IRC= -2.48155  Energy=  -169.968679130 a.u.
C    -0.021881    0.446923    0.000000
O    -1.123689   -0.055643    0.000000
N     1.079418   -0.334643    0.000000
H     0.165598    1.533057    0.000000
H     0.907338   -1.326883    0.000000
H     2.023568    0.005124    0.000000
  6
Point= -110  IRC= -2.27475  Energy=  -169.967083424 a.u.
C    -0.020319    0.460324    0.000000
O    -1.111973   -0.065040    0.000000
N     1.069005   -0.334290    0.000000
H     0.156170    1.547557    0.000000
H     0.854496   -1.318523    0.000000
H     2.025968   -0.033075    0.000000
  6
Point= -100  IRC= -2.06795  Energy=  -169.964896201 a.u.
C    -0.018552    0.473214    0.000000
O    -1.100345   -0.074322    0.000000
N     1.058758   -0.333716    0.000000
H     0.146321    1.561465    0.000000
H     0.800039   -1.307664    0.000000
H     2.027072   -0.071973    0.000000
  6
Point= -90  IRC= -1.86115  Energy=  -169.962069060 a.u.
C    -0.015995    0.485193    0.000000
O    -1.089196   -0.083506    0.000000
N     1.048859   -0.332788    0.000000
H     0.136340    1.574340    0.000000
H     0.740736   -1.292905    0.000000
H     2.026492   -0.109381    0.000000
  6
Point= -80  IRC= -1.65435  Energy=  -169.958473126 a.u.
C    -0.011819    0.495707    0.000000
O    -1.079357   -0.092539    0.000000
N     1.039882   -0.331594    0.000000
H     0.126487    1.585558    0.000000
H     0.671984   -1.271784    0.000000
H     2.023950   -0.140133    0.000000
  6
Point= -70  IRC= -1.44756  Energy=  -169.953762279 a.u.
C    -0.005634    0.504097    0.000000
O    -1.071893   -0.101079    0.000000
```

```
N     1.032768   -0.330537    0.000000
H     0.117189    1.594279    0.000000
H     0.591990   -1.242068    0.000000
H     2.019979   -0.157622    0.000000
  6
Point= -60  IRC= -1.24077  Energy=  -169.947392182 a.u.
C     0.001867    0.510093    0.000000
O    -1.067066   -0.108673    0.000000
N     1.027995   -0.329924    0.000000
H     0.108940    1.600079    0.000000
H     0.504739   -1.204672    0.000000
H     2.015888   -0.160193    0.000000
  6
Point= -50  IRC= -1.03397  Energy=  -169.938982257 a.u.
C     0.009673    0.514032    0.000000
O    -1.064151   -0.115225    0.000000
N     1.025259   -0.329523    0.000000
H     0.101870    1.603383    0.000000
H     0.413684   -1.164628    0.000000
H     2.012828   -0.152031    0.000000
  6
Point= -40  IRC= -0.82718  Energy=  -169.928564937 a.u.
C     0.017120    0.516430    0.000000
O    -1.062332   -0.120860    0.000000
N     1.024205   -0.328764    0.000000
H     0.095880    1.604947    0.000000
H     0.318265   -1.129690    0.000000
H     2.011325   -0.138206    0.000000
  6
Point= -30  IRC= -0.62038  Energy=  -169.916808876 a.u.
C     0.023861    0.517799    0.000000
O    -1.061066   -0.125729    0.000000
N     1.024557   -0.327353    0.000000
H     0.090820    1.605494    0.000000
H     0.218418   -1.104485    0.000000
H     2.010978   -0.122573    0.000000
  6
Point= -20  IRC= -0.41358  Energy=  -169.905527456 a.u.
C     0.029931    0.518596    0.000000
O    -1.060046   -0.130079    0.000000
N     1.025870   -0.325488    0.000000
H     0.086418    1.605564    0.000000
H     0.116153   -1.086465    0.000000
H     2.010944   -0.107032    0.000000
  6
Point= -10  IRC= -0.20678  Energy=  -169.897314805 a.u.
C     0.035579    0.519117    0.000000
O    -1.059126   -0.134174    0.000000
N     1.027711   -0.323460    0.000000
H     0.082360    1.605456    0.000000
H     0.013064   -1.070864    0.000000
H     2.010670   -0.091926    0.000000
  6
Point= 0  IRC=  0.00000  Energy=  -169.894301419 a.u.
```

```
C     0.000000    0.509746    0.000000
O    -1.099333   -0.147985    0.000000
N     0.988617   -0.331290    0.000000
H     0.036873    1.595627    0.000000
H    -0.131137   -1.064739    0.000000
H     1.968609   -0.086449    0.000000
  6
Point= 10  IRC=  0.20677  Energy=  -169.897241253 a.u.
C     0.046479    0.519767    0.000000
O    -1.057582   -0.142207    0.000000
N     1.032105   -0.319403    0.000000
H     0.074703    1.605105    0.000000
H    -0.192897   -1.036675    0.000000
H     2.008950   -0.062386    0.000000
  6
Point= 20  IRC=  0.41356  Energy=  -169.905160260 a.u.
C     0.052176    0.519883    0.000000
O    -1.057172   -0.146183    0.000000
N     1.034527   -0.317380    0.000000
H     0.070679    1.604889    0.000000
H    -0.295411   -1.018390    0.000000
H     2.007493   -0.046849    0.000000
  6
Point= 30  IRC=  0.62036  Energy=  -169.915457448 a.u.
C     0.058513    0.519634    0.000000
O    -1.057471   -0.149880    0.000000
N     1.037270   -0.315198    0.000000
H     0.066060    1.604504    0.000000
H    -0.397733   -1.004356    0.000000
H     2.005601   -0.029162    0.000000
  6
Point= 40  IRC=  0.82715  Energy=  -169.924977573 a.u.
C     0.066021    0.518376    0.000000
O    -1.059370   -0.152570    0.000000
N     1.040845   -0.312566    0.000000
H     0.060294    1.603457    0.000000
H    -0.498488   -1.004560    0.000000
H     2.003195   -0.006808    0.000000
  6
Point= 50  IRC=  1.03394  Energy=  -169.932293476 a.u.
C     0.074382    0.514834    0.000000
O    -1.063734   -0.153165    0.000000
N     1.046091   -0.309306    0.000000
H     0.053295    1.600510    0.000000
H    -0.591859   -1.024177    0.000000
H     2.000391    0.022072    0.000000
  6
Point= 60  IRC=  1.24073  Energy=  -169.937732067 a.u.
C     0.082034    0.508051    0.000000
O    -1.070379   -0.151581    0.000000
N     1.053846   -0.305596    0.000000
H     0.046108    1.594276    0.000000
H    -0.675332   -1.048220    0.000000
H     1.997634    0.056424    0.000000
```

```
  6
Point= 70  IRC=  1.44753  Energy=  -169.941809727 a.u.
C     0.087697    0.498110    0.000000
O    -1.078808   -0.148199    0.000000
N     1.064481   -0.301701    0.000000
H     0.040034    1.584568    0.000000
H    -0.748361   -1.065568    0.000000
H     1.995333    0.094055    0.000000
  6
Point= 80  IRC=  1.65432  Energy=  -169.944928712 a.u.
C     0.091123    0.485904    0.000000
O    -1.088401   -0.143488    0.000000
N     1.077402   -0.297793    0.000000
H     0.035854    1.572440    0.000000
H    -0.810416   -1.075624    0.000000
H     1.993476    0.132516    0.000000
  6
Point= 90  IRC=  1.86112  Energy=  -169.947300264 a.u.
C     0.093012    0.472429    0.000000
O    -1.098598   -0.138038    0.000000
N     1.091600   -0.293868    0.000000
H     0.033499    1.559047    0.000000
H    -0.864091   -1.080736    0.000000
H     1.991573    0.170433    0.000000
  6
Point= 100  IRC=  2.06792  Energy=  -169.949009393 a.u.
C     0.094203    0.458350    0.000000
O    -1.109094   -0.132301    0.000000
N     1.106350   -0.289857    0.000000
H     0.032442    1.545088    0.000000
H    -0.913166   -1.082961    0.000000
H     1.989135    0.207466    0.000000
  6
Point= 110  IRC=  2.27473  Energy=  -169.950096381 a.u.
C     0.095224    0.444029    0.000000
O    -1.119750   -0.126530    0.000000
N     1.121308   -0.285714    0.000000
H     0.032072    1.530909    0.000000
H    -0.960500   -1.083431    0.000000
H     1.985970    0.243501    0.000000
```
